# Determinants of vaccination coverage in rural Nigeria

**DOI:** 10.1186/1471-2458-8-381

**Published:** 2008-11-05

**Authors:** Olumuyiwa O Odusanya, Ewan F Alufohai, Francois P Meurice, Vincent I Ahonkhai

**Affiliations:** 1 Department of Community Health & Primary Health Care, Lagos State University College of Medicine, P.M.B. 21216, Ikeja, Lagos State, Nigeria; 2 Department of Surgery, College of Medicine, Ambrose-Alli University, Ekpoma, Edo state, Nigeria; 3 GlaxoSmithKline Biologicals, Rue de l'Institut 89, 1330 Rixensart, Belgium; 4 GlaxoSmithKline Biologicals, North American Vaccine Development, 2301 Renaissance Blvd, King of Prussia, PA 19406; USA

## Abstract

**Background:**

Childhood immunization is a cost effective public health strategy. Expanded Programme on Immunisation (EPI) services have been provided in a rural Nigerian community (Sabongidda-Ora, Edo State) at no cost to the community since 1998 through a privately financed vaccination project (private public partnership). The objective of this survey was to assess vaccination coverage and its determinants in this rural community in Nigeria

**Methods:**

A cross-sectional survey was conducted in September 2006, which included the use of interviewer-administered questionnaire to assess knowledge of mothers of children aged 12–23 months and vaccination coverage. Survey participants were selected following the World Health Organization's (WHO) immunization coverage cluster survey design. Vaccination coverage was assessed by vaccination card and maternal history. A child was said to be fully immunized if he or she had received all of the following vaccines: a dose of Bacille Calmette Guerin (BCG), three doses of oral polio (OPV), three doses of diphtheria, pertussis and tetanus (DPT), three doses of hepatitis B (HB) and one dose of measles by the time he or she was enrolled in the survey, i.e. between the ages of 12–23 months. Knowledge of the mothers was graded as satisfactory if mothers had at least a score of 3 out of a maximum of 5 points. Logistic regression was performed to identify determinants of full immunization status.

**Results:**

Three hundred and thirty-nine mothers and 339 children (each mother had one eligible child) were included in the survey. Most of the mothers (99.1%) had very positive attitudes to immunization and > 55% were generally knowledgeable about symptoms of vaccine preventable diseases except for difficulty in breathing (as symptom of diphtheria). Two hundred and ninety-five mothers (87.0%) had a satisfactory level of knowledge. Vaccination coverage against all the seven childhood vaccine preventable diseases was 61.9% although it was significantly higher (p = 0.002) amongst those who had a vaccination card (131/188, 69.7%) than in those assessed by maternal history (79/151, 52.3%). Multiple logistic regression showed that mothers' knowledge of immunization (p = 0.006) and vaccination at a privately funded health facility (p < 0.001) were significantly correlated with the rate of full immunization.

**Conclusion:**

Eight years after initiation of this privately financed vaccination project (private-public partnership), vaccination coverage in this rural community is at a level that provides high protection (81%) against DPT/OPV. Completeness of vaccination was significantly correlated with knowledge of mothers on immunization and adequate attention should be given to this if high coverage levels are to be sustained.

## Background

Immunization remains one of the most important public health interventions and a cost effective strategy to reduce both the morbidity and mortality associated with infectious diseases. Over two million deaths are delayed through immunization each year worldwide [1]. Despite this, vaccine preventable diseases remain the most common cause of childhood mortality with an estimated three million deaths each year [2]. Uptake of vaccination services is dependent not only on provision of these services but also on other factors including knowledge and attitude of mothers [3,4], density of health workers [5], accessibility to vaccination clinics and availability of safe needles and syringes.

Assessing immunization coverage helps to evaluate progress in achieving programme objectives and in improving service delivery [6]. In addition, evaluation of immunization coverage provides evidence whether substantial progress towards achieving vaccination targets is being made. Such positive evidence is required for continuing support from donor-supported initiatives like the Global Alliance for Vaccines and Immunizations (GAVI) [7].

This paper reports on a survey assessing vaccination coverage for childhood vaccines and maternal factors impacting coverage in a rural community, Sabongidda-Ora in Edo State, mid-western Nigeria. Since 1998 the area has been receiving free vaccination services from a privately financed vaccination project with government support established (as a public-private partnership) by GlaxoSmithKline (GSK) Biologicals. Childhood vaccines provided under the auspices of this project are Bacille Calmette Guerin (BCG), diphtheria, pertussis and tetanus (DPT), measles, oral polio (OPV), hepatitis B (HB) and yellow fever. At the GSK supported health facility health education is given top priority. In addition, it provides care for minor childhood illnesses. The authors have previously reported on the socio-demographic characteristics of the community [8]. The project was established at a time immunization coverage in Nigeria had dropped drastically and vaccines were not regularly available [9]. For the first few years when the project started offering services, it was the only source of vaccination in the community. The immunization coverage (BCG, OPV3, DPT3 and measles) at the inception of the project was 43% [10] but rose rapidly to 78% after 2 years, and hepatitis B coverage (three doses) was implemented and reached 58% in 2000 [8].

The main immunization facilities in the project area at the time of the survey are the GSK supported Health Centre, government health centres and a few private (fee for profit) clinics. The GSK supported Health Centre and the main government health centre are located within the same area of Sabongida-Ora (see additional file 1) The private clinics receive their vaccine supplies from government health centres, and services from both sources have moderately improved in recent years. The Nigerian National Programme on Immunization (NPI) schedule is BCG, OPV0, HB1 (first dose) at birth; DPT1, OPV1, HB 2 (second dose) at 6 weeks; DPT2, OPV2 at 10 weeks; DPT3, OPV3 HB3 (third dose) at 14 weeks; measles, and yellow fever at 9 months. The GSK supported Health Centre follows this schedule but uses a combined DPT/HB vaccine, with three doses of the combined vaccine being given. As the combined vaccine cannot be given at birth, an additional single dose HB vaccine is given at birth.

The project has continued to offer childhood vaccination services to date, it was in its eighth year of operation at the time of this survey and the last assessment of immunization coverage was conducted six years earlier [8]. Thus it was decided to assess the immunization coverage of children aged 12–23 months in the area and to identify the determinants of full immunization status. This is likely to help in identifying maternal characteristics that could be predictive of immunization coverage and lead to improvement in services.

## Methods

### Survey design

The survey was conducted as a community-based cross sectional descriptive survey. The survey involved interviewing the mothers of eligible children using a protocol to obtain maternal characteristics and immunization history. Selection of mothers for interview was done following World Health Organization's (WHO') immunization coverage cluster survey design.

To be eligible for recruitment into the survey, the mother/caregivers were required to give consent, live in and around Sabongidda-Ora and have children who were born between 01 October 2004 and 31 August 2005 (12–23 months old). The survey was conducted in September 2006.

### Sample size determination

Using the sample size calculation methodology presented in the WHO Immunization Coverage Cluster Survey Reference Manual (WHO/IVB/04.23), the sample size required was determined using the coverage of 78% obtained in the year 2000 [8], a precision of ± 6.5%, a type 1 error of 5% and a design effect of 2, in conformity with the standard WHO methodology [11]. Thus the calculated number of children required was 312.

### Survey instrument

An interviewer-administered questionnaire was used to obtain data. The instrument sought for socio-demographic information of mothers, knowledge of vaccination schedule, knowledge about vaccine-preventable diseases, history of vaccines received by the child and reasons for non-vaccination. Most of the questions on the survey instrument were close-ended. In addition, information about the child's vaccination was obtained from the vaccination card (where available) or by maternal history and transferred to the survey instrument. The protocol was constructed from a review of available literature on immunization coverage [3,4,9,10].

### Data collection

Four main communities (Sabongidda, Oke, Aviobsi and Uhonmora, see additional file 2) within the catchment areas of the project site were visited during the survey. Households in the communities were visited by trained data collectors to identify mothers with eligible children. Such children were recruited consecutively until the sample size was achieved. The first household in each community was selected using the spinning bottle method and the direction was followed until all the households were exhausted. Data quality was ensured through onsite supervision and review of completed forms. Mothers who consented to participate in the survey received one bottle of a multivitamin preparation as an incentive.

### Data analysis

Each completed subject survey form was reviewed for completeness prior to analysis by the principal investigator. Subjects whose ages were outside the eligible age group and those with incomplete vaccination records were excluded from data analysis.

The knowledge of the mothers was scored on a five point scale. Each domain was scored one point for correct response and zero for wrong answers. The points consisted of ability to correctly state the purpose of immunization (this was an open ended question, assessed by the principal investigator, and the correct answer was to protect against communicable diseases), knowledge of the right age a child should receive measles vaccine, and the number of times oral polio should be received. The other domains were knowledge of the age at which a child should complete his/her vaccinations and knowledge of at least three symptoms of vaccine preventable illnesses. The symptoms of interest were cough, skin rash, jaundice, paralysis, very high fever and difficulty in breathing. Satisfactory knowledge was classified as a minimum score of three points. This scoring system is similar to that reported by some other researchers [3].

In determining the validity of immunizations, the doses must have been given at the recommended ages and for multiple dose antigens, not less than four weeks intervals between the doses. This was done by evaluating the dates of the vaccinations recorded on the immunization cards and by careful history taking from those without cards. The hepatitis B (HB) doses given at the GSK supported Health Centre and Government facilities were harmonized for the purposes of data analysis. For subjects who were vaccinated at the GSK supported Health Centre, HB1 was taken as HB dose at birth, HB2 was HB given at 6 weeks and HB3 was HB given at 10 or 14 weeks. This was consistent with the Nigerian NPI schedule. The primary assessment of HB coverage was uptake of at least three HB doses. A child was regarded as fully immunized if he/she received a dose of BCG, three doses of OPV & DTP, three doses of HB and measles vaccine [4].

All validly completed forms were entered into a computer and analysed with EpiInfo software v.6.04c. Double data entry was performed and validated. Data were cleaned and several consistency checks carried out to ensure accuracy and completeness. Both descriptive and inferential statistics were computed. Simple frequency tables of maternal and child characteristics were made. The confidence level was set at 95%. Chi square test was conducted to explore the association between maternal/caregiver characteristics, other variables and completion of immunization. Factors that were found to be significant at p < 0.05 from the bivariate analysis were entered into a multiple logistic regression model.

### Ethics approval

Ethics approval was obtained from the ethics Committee of the Ambrose Alli University College of Medicine and also from the community leaders.

## Results

### Socio-demographic characteristics of subjects

Three hundred and fifty three children were recruited into the survey. However, 14 (4.0%) were excluded from further analysis; 10 due to being ineligible on account of age (6 were over age and 4 were under age), and four on account of incompletely filled survey forms. Thus 339 (96.1%) subjects were analyzed, with each mother having only one eligible child. Recruitment of subjects from the main communities was Sabongidda (196), Oke (51), Aviobsi (73) and Uhonmora (19) children respectively.

Table 1 shows the socio-demographic characteristics of the mothers/caregivers and children. The mean age (± standard deviation) of the mothers/caregivers was 29.9 ± 5.8 years. The majority were married (95%) and were of the Christian faith (93%, this part of Nigeria is predominantly a Christian community). The age of the mothers did not show any significant variation across the four communities (p = 0.28). There were no significant relationships between age and marital status (p = 0.14) and religion (p = 0.29). Two hundred and twelve (62.5%) had obtained at least a secondary school level of education (at least 12 years or more of school) and almost half (48.4%) were engaged in petty trading. The mothers had a mean of 3.0 ± 1.7 children. The mean age of the children was 17.5 ± 3.5 months, and 185 (55%) were females.

**Table 1:** Socio-demographic Characteristics of Mothers and Children in Rural Nigeria

**Variable**	**N**	**%**
**Age group (years)**		
< 20	15	4.4
20–29	197	58.1
30–39	108	31.9
40–49	13	3.8
≥ 50	1	0.3
Unstated	5	1.5
	339	100
**Marital Status**		
Single	15	4.4
Married	321	94.7
Divorced/separated	3	0.9
	339	100
**Religion**		
Christianity	314	92.6
Islam	22	6.5
African Traditional	3	0.9
	339	100
**Level of Education**		
None	14	4.1
Primary	93	27.4
Secondary	214	63.1
University/polytechnic	18	5.3
	339	100
**Occupation**		
Housewife/unemployed	69	20.4
Trader	165	48.7
Artisan	73	21.5
Skilled workers	11	3.2
Professionals	8	2.4
Others	13	3.8
	339	100
**No of children**		
1	78	23.0
2–4	195	57.5
≥ 5	66	19.5
	339	100
Mean age ± SD (months)of children	17.5 ± 3.5	
Gender of children		
Females	185	54.6
Males	154	45.4

### Knowledge and attitudes on immunization

The knowledge and attitudes of the mothers/care givers towards immunization are shown on Table 2. Most mothers had very positive attitudes and more than half of them were generally knowledgeable about symptoms of vaccine preventable diseases except for difficulty in breathing. Cough was the most correctly identified symptom (83.8%) and almost all (99.1%) felt that immunization was beneficial. The mean score was 3.9 ± 1.2 points with 295 (87.0%) of the mothers scoring above 3 marks and were classified as having a satisfactory knowledge on the aspects inquired. Age (p = 0.38), marital status (p = 0.09) and place of residence (p = 0.09) were not significantly associated with higher level of knowledge. However, the knowledge of the mothers was significantly higher with being of the Christian faith (p = 0.002) and possessing at least secondary school education (p = 0.009). Further analysis of the effect of education on the knowledge scores stratified by religion did not show any significant association (Mantel-Haenszel summary Chi Square = 0.82; p = 0.364) indicating that religion was a confounding variable for the influence of education.

**Table 2:** Attitudes and Knowledge of Mothers in Rural Nigeria on some aspects of Immunization

**Variable**	**N (339)**	**%**
Feels that Immunization is beneficial	336	99.1
Would advise other mothers to get their children immunized	336	99.1
Correct definition/purpose of immunization	216	63.7
Correct knowledge of number of times oral polio should be received	254	74.9
Correct knowledge of the age at which measles vaccine is received	290	85.5
Correct knowledge that vaccination schedule should be completed before 12 months	304	89.7
**Knowledge of vaccine preventable Symptoms**		
Cough (Tuberculosis/Pertussis)	284	83.8
Difficulty in breathing (Diphtheria)	122	36.0
Skin rash (measles)	211	62.2
Paralysis (Poliomyelitis)	244	72.0
Jaundice (yellow fever/hepatitis B)	189	55.8
High fever (Pertussis)	209	61.7

### Vaccination status of children

Three hundred and seventeen (93.5%) children from whom vaccination cards or verbal immunization history was verified had received at least one antigen, 22 (6.5%) had not received any vaccine at all. The card retention rate for all subjects was 55.5%. The majority (68.6%) were vaccinated at the GSK supported Health Centre. Vaccination coverage is shown on Table 3. The DPT1 drop-out rate was 8.0%. DPT3 coverage was 80.8%. Six out of ten children were fully vaccinated against tuberculosis, tetanus, diphtheria, poliomyelitis, pertussis, measles and hepatitis B, although the coverage was significantly higher (p = 0.002) amongst subjects who had vaccination cards (69.7%, 131/188) than in those assessed by maternal history alone (52.3%, 79/151). Comparing maternal assessment of completeness of vaccination with full immunization status showed that this was correct in 209/210 of those fully vaccinated and in 61/129 of those not fully vaccinated. The sensitivity of maternal history was 99.5% (Confidence Interval (CI) 97,100%), the specificity (when mothers do not truly know the vaccination status of their children) was 47.3% (CI 38.5, 56.2%), positive predictive value of 75.5 (CI 69.9, 80.3%) and negative predictive value of 98.4% (CI 90.2, 99.9%). The confidence intervals for the sensitivity, specificity, positive predictive value and negative predictive value were scores intervals. The percentage of correct classification is 79.6%.

**Table 3:** Vaccination Status of Children in Rural Nigeria by Place of Vaccination

**Variable**	**Place of vaccination (%)**			**Total**
	GSK site (n = 231)	Govt (n = 81)	Private (n = 27)	(n = 339)
BCG	100	97.5	14.8	92.6
Oral Polio at birth	41.1	60.5	3.7	43.8
DPT1/OPV1	99.1	86.4	7.4	88.8
DPT2/OPV2	97.4	74.1	7.4	84.7
DPT3/OPV3	94.8	64.2	11.1	80.8
Measles	85.3	66.7	3.7	74.3
Yellow fever	60.6	40.7	3.7	51.3
Three doses of HB vaccine	94.8	64.2	11.1	80.8
Vaccinated against all 7 diseases	82.3	18.5	0.0	61.9

The proportion of children fully vaccinated at the GSK supported Health Centre (190/231, 82.3%) was significantly higher (p < 0.001) than the rate observed for children vaccinated at the government/private facilities (20/108, 18.5%). The proportion of children that were fully vaccinated amongst the four communities was Sabongidda 68.4%, Oke 66.7%, Aviobsi 45.2% and Uhonmora 47.4%. Maternal factors were most strongly associated with non-completion of vaccination; the most frequent was lack of awareness of the need for immunization.

Factors that were associated with a higher rate of immunization against all the seven diseases included being from Sabongidda (p = 0.006), having at least secondary school education (p = 0.035), satisfactory immunization knowledge of the mother (p < 0.001), retention of immunization card (p = 0.002) and vaccination at the GSK supported Health Centre (p < 0.001, Table 4). Table 5 reveals the results of the multiple logistic regression. Vaccination at the GSK supported Health Centre (p < 0.001) and satisfactory maternal knowledge on immunization (p = 0.006) were the only factors that were significantly associated with completion of vaccination. The estimates of the knowledge scores and maternal education were in opposite directions. This is probably related to the influence of maternal education as a confounding variable on the knowledge scores.

**Table 4:** Bivariate statistical analysis – Completion of Vaccination in rural Nigeria

**Variable**	**N**	**%**	**X**^2^	**P-value**
**Community**				
Sabongidda-Ora	134/196	68.4		
Other communities	76/143	53.1	7.49	0.006
**Marital status**				
Single/divorced	10/18	55.6		
Married	200/321	62.3	0.11	0.746
**Religion**				
Christianity	198/314	63.1		
Islam/Traditional	12/25	48.0	1.63	0.201
**Education**				
None/Primary	57/107	63.1		
Secondary/University	153/232	65.9	4.47	0.035
**Age**				
≤ 29 years	123/217	56.7		
≥ 30 years	83/122	68.0	2.60	0.107
**Knowledge Score**				
Poor	14/44	31.8		
Satisfactory	196/295	66.4	18.03	< 0.001
**Gender**				
Female	110/185	59.5		
Male	100/154	64.9	0.85	0.357
**Age group of Child**				
12–17 months	109/175	62.3		
18–23 months	101/164	61.6	0.00	0.983
**Retention of Immunization card**				
Yes	131/188	69.7		
No	79/151	52.3	9.99	0.002
**Place of Vaccination**				
GSK supported Health Centre	190/231	82.2		
Government/Private	20/108	18.5	124.12	< 0.001

**Table 5:** Multiple logistic regression of Determinants of Completion of Vaccination in rural Nigeria

	**Odds Ratio**			
**Variable**	**point estimate**	**95%CI**		**P-value**
**Community**				
Sabongidda-Ora	1.63	0.85	3.11	0.145
Other communities	1			
**Education**				
None/Primary	1			
Secondary/University	1.79	0.97	3.31	0.064
**Knowledge Score**				
Poor	1			
Satisfactory	0.31	0.13	0.72	0.006
**Retention of Immunization card**				
Yes	0.60	0.31	1.17	0.134
No	1			
**Place of Vaccination**				
GSK	30.39	14.12	65.42	< 0.001
Government/Private	1			

## Discussion

### Knowledge and attitudes of mothers on immunization

The level of education of mothers recruited into this survey was high and may not be usual for rural communities. However, it is not unexpected for this part of Nigeria. Edo state used to be part of the defunct western Region of Nigeria, which introduced free and compulsory primary education in 1955 and has continued to be one of the states with the highest primary school enrolment rates in the country. The knowledge and attitudes of the mothers participating in this survey were found to be high and generally positive. Two hundred and ninety-five (87.0%) possessed a satisfactory level of knowledge. The significantly higher level of knowledge found amongst mothers with secondary and higher level of education is as expected as they ought to understand scientific information more easily than those with lower level of education. This high level of knowledge may be attributable to the quality of information provided to mothers at the health facilities. The level of awareness in this community contrasts with a low rate (4%) of knowledge about OPV reported from Niger [12] and an equally low rate of awareness (1%) that measles was preventable by immunization from Nigeria [13] but comparable to the 97% of survey participants who were able to define immunization in Nairobi, Kenya [14]. However, in Niger, the vaccination coverage against polio was higher than in our survey area despite the low level of awareness by the mothers in that country due to more frequent use of "National Polio Immunization Days" [12]. Furthermore, the knowledge of mothers in our community on the symptoms of vaccine-preventable diseases and vaccination schedules was good compared to a report from India where such knowledge was very limited save for poliomyelitis [15].

### Vaccinations status and determinants

The vaccination coverage presented in this survey reflects the vaccination status of the children as at August 2006. The immunization coverage for BCG, DPT, and OPV was above 80%. Because of the long interval between the third dose of DTP and measles, a number of children do not return for measles vaccine and this makes the coverage rate for this antigen to be lower than others in keeping with the reported pattern [10,16]. The proportion of children fully vaccinated was just over 60%. To our knowledge, this survey presents the first documented coverage rate for yellow fever vaccine (51%) in Nigeria, a new addition to the EPI antigens which hitherto was given only for epidemic outbreak control.

The coverage rates in this survey (BCG 93%, DPT3/OPV3 81%, HB3 81%, measles 74%) are higher than the WHO-UNICEF estimates for Nigeria (BCG 69%, DPT3 54%, OPV3 61%, HB3 41%, measles 62%) [17]. However, they are comparable with rates from Delhi, India (BCG 82%, DPT3 70%, measles 66%) [16], and with the WHO-UNICEF estimates for Ghana (BCG 99%, DPT3/OPV3 84%, HB3 84%, measles 85%) [18]. The higher coverage rates amongst children who received their vaccines at the GSK supported health facility may be a reflection of continuing patronage and confidence amongst the mothers in the facility. In comparison with the year 2000 survey [8], a higher but not significant proportion (55% vs. 62%, p = 0.06) of children were fully vaccinated. However, the coverage rates in this present survey were higher than the rates that were found at the start of the project in 1998 [10]. The coverage was expected to be much higher than was found in the present survey given the momentum and initial rapid rise in coverage found in the initial two years of project. In this present survey, there was a sharp decline in coverage rates from BCG (93%) at birth, to measles (74%) and the proportion that was fully vaccinated (62%) indicating that many of the children are lost to follow up in later months and some (who take the later antigens) skip some of the vaccines. The lower than expected coverage rate observed in this survey reinforces the need for continuous staff motivation, regular supervision and continuous monitoring and evaluation to detect declines in vaccination coverage very early.

An important finding from this survey is the sensitivity and accuracy of maternal recall of adequacy of vaccination showing it may be possible to rely on maternal history to determine vaccination coverage in this community although the specificity (when mothers do not know) is low. The sensitivity and accuracy of maternal recall obtained in this community is much higher than has been reported from India [19]. Besides, other studies have reported a close relationship between vaccination coverage obtained by vaccination card and maternal history [20].

The significant predictors of completeness of immunization found on bivariate analysis such as maternal education and retention of immunization card found in this survey have been confirmed by other researchers [16]. Logistic regression identified maternal knowledge and vaccination at the GSK supported Health Centre were significantly correlated with the rate of full immunization. This role of maternal knowledge as an important determinant of vaccination coverage has been shown by several researchers [3,4,13] even in communities with a high level of illiteracy [21]. Therefore it is possible to state that the findings of the study may be generalisable to at least Edo State of Nigeria. However, this link is more important when vaccines are routinely administered in permanent health care structures, as opposed to National Immunization Days. Some other workers have reported that vaccination coverage is higher in areas served by private agencies than government facilities [22], a finding similar to the results in this survey. It is important to observe that 6.5% of the children recruited in this survey had not been vaccinated at all. This was mainly amongst temporary inhabitants and new arrivals in the community. The difficulty in ensuring adequate vaccination amongst migrant populations has been reported from Niger [11]. The major reasons (lack of awareness of the need for immunization) adduced for failure to be fully vaccinated are as reported from Niger [11].

### Limitations of this Survey

Nigeria has conducted several supplemental vaccination activities in many states including Edo State where this project is situated. These supplemental activities were typically national and state immunization days for oral polio vaccine. Thus the estimates of OPV reported herein may not be solely from our vaccination facilities and the contribution of these activities could not be estimated. However coverage rates for OPV3 were similar to DTP3 (81%). The 56% card retention rate (which is lower than the last survey in this community 81% [8]) is a constraint to obtaining a very accurate assessment of immunization coverage. However, it is accepted practice to estimate immunization coverage by maternal history in both developing [20] and developed nations [23]. The survey did not explore other factors such as paternal and family characteristics that may have significant association with children being fully immunized.

## Conclusion

Three hundred and thirty-nine mothers were included in this survey; 295 (87%) had a satisfactory level of knowledge on immunization. Sixty two percent of the eligible children were fully vaccinated, which was slightly higher than the 55% coverage rate reported in the year 2000 survey [8]. Important correlates of full vaccination were maternal education and vaccination at the GSK supported Health Centre. Recommendations for improving immunization coverage in this community include regular supervision, more frequent but limited assessment of vaccination coverage and continuous education of women at the health facilities. Increased enrolment of girls into secondary school should be generally encouraged and is likely to have a favourable impact on vaccination rates of their future offspring. To strengthen the accuracy of vaccination coverage estimates, more attention should be paid to immunization card retention.

## Competing interests

All authors have either been employees of and or have received compensation from GlaxoSmithKline Biologicals.

## Authors' contributions

OOO was the principal investigator, Study concept, study protocol writing, data analysis and manuscript writing. He will serve as guarantor for the study. The other authors (EA, FM, & VA) were jointly responsible for study concept and writing the manuscript. All authors have seen and approved the final version of the manuscript.

## Pre-publication history

The pre-publication history for this paper can be accessed here:



## Supplementary Material

Additional file 1 **FIGURE 1. Map of Sabongidda-Ora, Owan -west Local Government Area, Edo State, Nigeria**. This is a map of the survey area (Sabongidda-Ora) indicating that the GSK supported facility and the Government facility are in the same axis of the town. Click here for file

Additional file 2 **FIGURE 2. Map of Owan-west Local Government Area, Edo State, Nigeria**. This is a map of the Local Government Area showing the four communities where the survey took place. Click here for file
