# Supplementary material for: Determinants of vaccination coverage in rural Nigeria
Source: BMC Public Health. 2008 Nov 5;8:381. doi: 10.1186/1471-2458-8-381 (PMC2587468; doi:10.1186/1471-2458-8-381)
Supplement: Additional file 1 — FIGURE 1. Map of Sabongidda-Ora, Owan -west Local Government Area, Edo State, Nigeria. This is a map of the survey area (Sabongidda-Ora) indicating that the GSK supported facility and the Government facility are in the same axis of the town. [file 1471-2458-8-381-S1.pdf]

FIGURE 1. MAP OF SABONGIDDA-ORA, OWAN-WEST LOCAL GOVERNMENT AREA, EDO STATE, NIGERIA

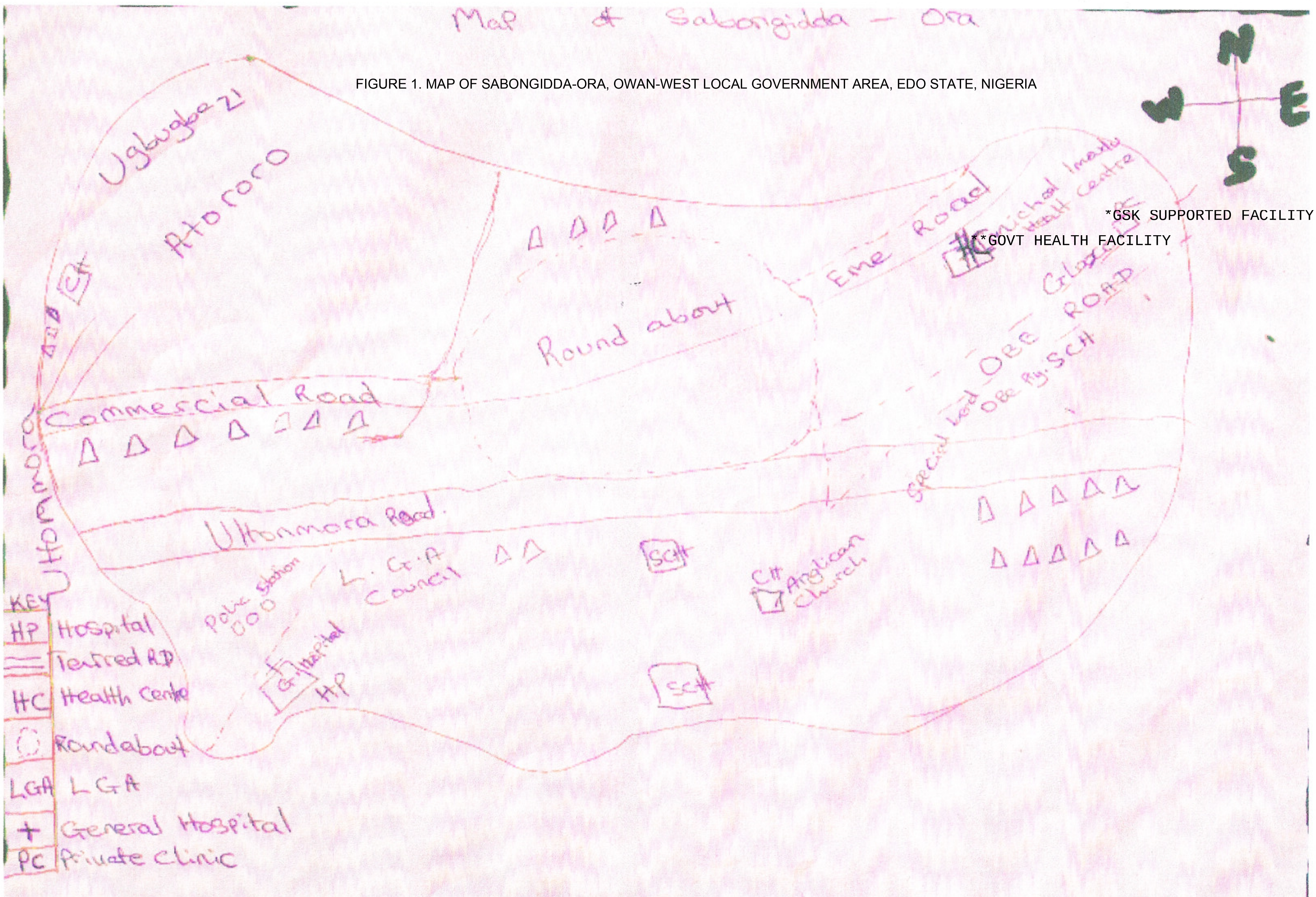

*GSK SUPPORTED FACILITY

**GOVT HEALTH FACILITY
