# Supplementary figures and images for: Determinants of vaccination coverage in rural Nigeria
Source: BMC Public Health. 2008 Nov 5;8:381. doi: 10.1186/1471-2458-8-381 (PMC2587468; doi:10.1186/1471-2458-8-381)

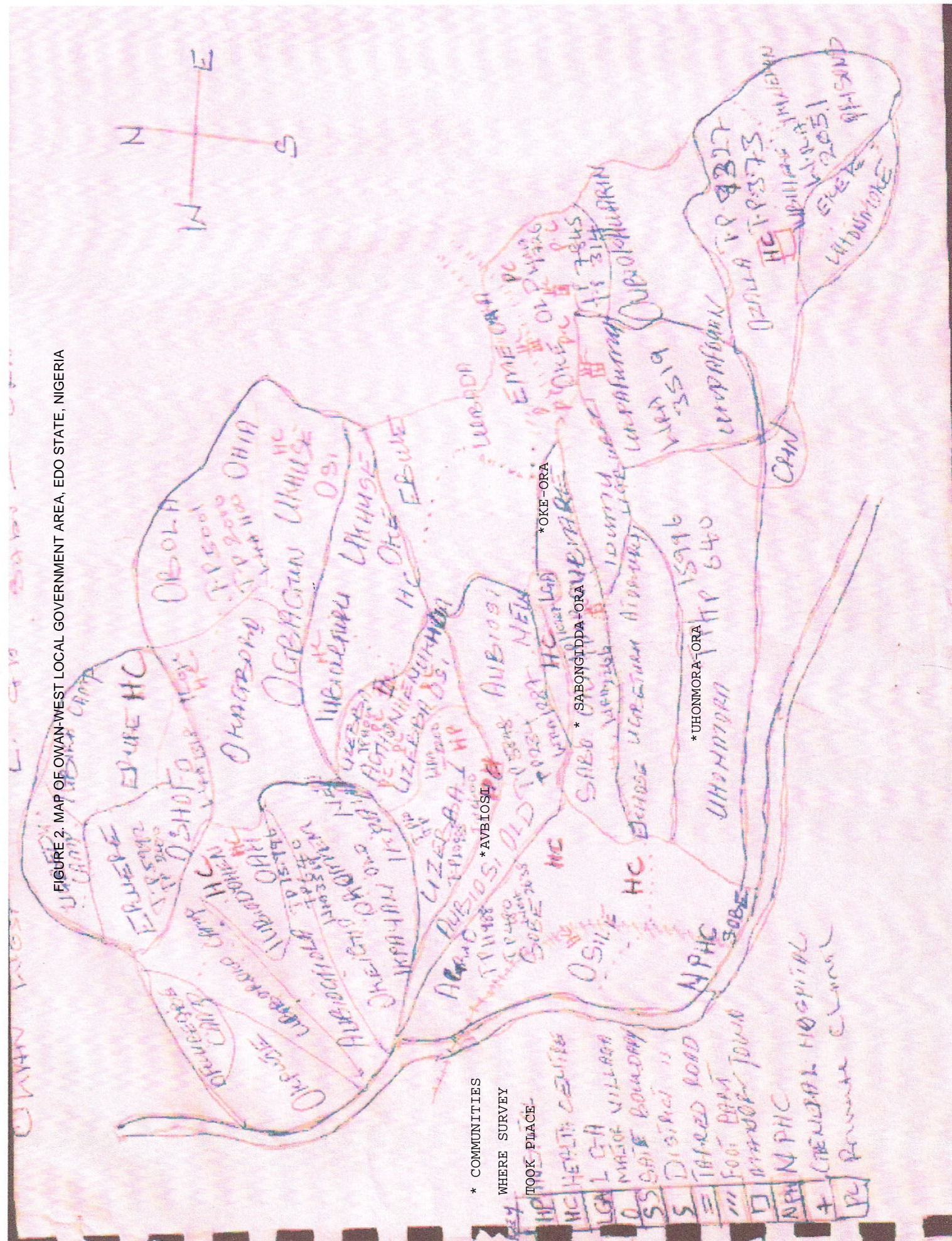

Supplement: Additional file 2 — FIGURE 2. Map of Owan-west Local Government Area, Edo State, Nigeria. This is a map of the Local Government Area showing the four communities where the survey took place. [file 1471-2458-8-381-S2.pdf]
